# The organellar genomes of Pellidae (Marchantiophyta): the evidence of cryptic speciation, conflicting phylogenies and extraordinary reduction of mitogenomes in simple thalloid liverwort lineage

**DOI:** 10.1038/s41598-023-35269-3

**Published:** 2023-05-23

**Authors:** Łukasz Paukszto, Piotr Górski, Katarzyna Krawczyk, Mateusz Maździarz, Monika Szczecińska, Monika Ślipiko, Jakub Sawicki

**Affiliations:** 1 Department of Botany and Nature Protection, University of Warmia and Mazury in Olsztyn, Plac Łódzki 1, 10-727 Olsztyn, Poland; 2 Department of Botany, Poznań University of Life Sciences, ul. Wojska Polskiego 71C, 60-625 Poznań, Poland; 3 Department of Ecology and Environmental Protection, University of Warmia and Mazury in Olsztyn, Plac Łódzki 3, 10-727 Olsztyn, Poland

**Keywords:** Computational biology and bioinformatics, Genetics, Molecular biology, Plant sciences

## Abstract

Organellar genomes of liverworts are considered as one of the most stable among plants, with rare events of gene loss and structural rearrangements. However, not all lineages of liverworts are equally explored in the field of organellar genomics, and subclass Pellidae is one of the less known. Hybrid assembly, using both short- and long-read technologies enabled the assembly of repeat-rich mitogenomes of *Pellia* and *Apopellia* revealing extraordinary reduction of length in the latter which impacts only intergenic spacers. The mitogenomes of *Apopellia* were revealed to be the smallest among all known liverworts—109 k bp, despite retaining all introns. The study also showed the loss of one *tRNA* gene in *Apopellia* mitogenome, although it had no impact on the codon usage pattern of mitochondrial protein coding genes. Moreover, it was revealed that *Apopellia* and *Pellia* differ in codon usage by plastome CDSs, despite identical *tRNA* gene content. Molecular identification of species is especially important where traditional taxonomic methods fail, especially within Pellidae where cryptic speciation is well recognized. The simple morphology of these species and a tendency towards environmental plasticity make them complicated in identification. Application of super-barcodes, based on complete mitochondrial or plastid genomes sequences enable identification of all cryptic lineages within *Apopellia* and *Pellia* genera, however in some particular cases, mitogenomes were more efficient in species delimitation than plastomes.

## Introduction

The species fundamental unit in systematic biology is not strictly defined and several species concepts function in parallel in science. No less than 24 definitions of species exist^1^ but the most common and popular definition of species is based on morphological differences^2^, however not all species can be defined and described that way. Often in the taxa with lower morphological complexity, the accumulation of genetic and ecological differences is not correlated with the accumulation of morphological variation, this situation leads to the rise of cryptic species. Cryptic species are characterised by different ecological preferences, pattern of geographic distribution, distinctive genetic differences, and the absence or minor morphological variation^3^.

The cryptic speciation of liverworts is a well studied phenomenon, and known from all evolutionary lineages of these organisms^4–7^. In recent years integrative taxonomy approach led to description of several species of liverworts, previously considered as cryptic lineages distinguishable only on the basis of molecular markers^8,9^. However, most of successfully morphologically characterised and described former cryptic taxa belong to lineages with more complex morphology, whereas the lower structural complexity of simple thalloid liverworts makes macro- and microscopic delimitation more challenging. Based on morphology, liverworts have been subdivided into an early divergent Haplomitriopsida, the Marchantiopsida (complex thalloids), and the Jungermanniopsida, which comprises two morphological subgroups: simple thalloids and leafy hepatics^10,11^. The first mentioned class, Haplomitriopsida, includes leafy plants, radially symmetric, with polystratose, identical leaves, arranged in three rows. Plants classified as Marchantiopsida develop thick thallus differentiated into dorsal zones (with air-chambers and air-pores) and ventral ones with storage tissue. The third group (Jungermanniopsida) comprises both leafy and thallose plants differing in distinctive features from those mentioned above. Leafy Jungermanniopsida (Jungermanniidae subclass) have unistatose and morphologically diverse (lateral and ventral) leaves, bilateral symmetry and differ in these respects from Haplomitriopsida. Simple thalloids (Pelliidae and Metzgeriidae subclasses) develop thin thallus (mainly unistatose) without air-chambers and pores. Both of these groups share many features. The most striking is polystratose thallus without air-chambers and presence of a central strand of dead, water conducting cells (known also as midrib). An important difference is the location of the gametangia. Gynoecia in Pelliidae are usually anacrogynous, on dorsal surface of midrib or stem (here are also antheridia), whereas gynoecia in Metzgeriidae are acrogynous, on abbreviated lateral or ventral branches (with antheridia). The group of Pelliidae is more taxonomically diverse (three orders and 21 genera), while the Metzgeriidae include two orders (and eight genera), but on a global scale, more species are described within the last group^12^.

Subclass Pelliidae includes both thalloid and leafy forms. The order Pelliales, which according to recent molecular phylogenies is the earliest diverging lineage within simple thalloid liverworts^13,14^, comprises two families: Noterocladaceae and Pelliaceae. The first family is monospecific, with leafy and South American *Noteroclada* *confluens* Taylor^15,16^. The second one, Pelliaceae, comprises two genera, *Apopellia* and *Pellia*, with fleshy thalli and an obscure defined midrib, with seven species restricted to cool and temperate region of Holarctic^12,15^. The order Fossombroniales includes plants with obliquely inserted succubous leaves in two ranks (Fossombroniaceae; the most numerous group within the order), and thallose with well- or ill-defined midrib (Allisoniaceae, Calyculariaceae, Petalophyllaceae, and Makinoaceae)^12,15,17^. The last order within Pelliidae (Pallaviciniales) comprises plants with prostrate thallus and well-defined midrib, with 1–2(4) central strands of elongate conducting cells. These thallose forms are found within four families that belong to this order: *Pallaviciniaceae* (with eight genera: *Pallavicinia,* *Jensenia,* *Podomitrium,* *Greeneothallus,* *Seppeltia,* *Symphyogyna,* *Symphyogynopsis,* *Xenothallus*), Hymenophytaceae (with one genus *Hymenophyton*), Moerckiaceae (*Hattorianthus*, *Moerckia*), and Sandeothallaceae (with one genus *Sandeothallus*)^12^. A complete exception within the Pallaviciniales is the Phyllothalliaceae family, which has leafy forms with the leaves opposite, distant to contiguous, and well defined internodes^11^. This family includes one genus (*Phyllothallus*) with two species occurring in the Southern Hemisphere^12,18^. The phylogenetic studies divide Pelliidae into three orders: Pelliales, Fossombroniales and Pallaviciniales, but monophylly of subfamily and evolutionary relationships among them are still a subject of discussion^19^. The order Pelliales, which according to recent molecular phylogenies is the earliest diverging lineage within simple thalloid liverworts^13,14^, including genera like *Apopellia* and *Pellia*, which are becoming model taxa for studying of the speciation mechanisms. The study of cryptic speciation of genus *Pellia* s.l. has a long history, starting with the description of polyploid *P.* *borealis* at the beginning of the twentieth century^20^. The development of the isoenzyme electrophoresis methods enabled discovery of cryptic evolutionary lineages within *Pellia* sensu lato^21,22^, which were later confirmed by various PCR based markers^23–25^. Recently, an integrative approach based on morphology and molecular dataset has enabled the split of the genus *Pellia* into *Apopellia* and *Pellia* sensu stricto^26^. The former comprises three taxonomic species, *A.* *apicola*, *A.* *megaspora* and *A.* *endiviifolia*, with different patterns of distribution. The European population of *A.* *endiviifolia* is diverging into two evolutionary lineages named A (typical form) and B (water form), which, besides molecular characters, differ in types of microhabitats^24–26^.

Despite recent advances in liverwort organellar genomics, the simply thalloid Pelliidae are poorly explored in that field. Chloroplast genome of *Apopellia* *endiviifolia* was among the first sequenced liverwort plastomes^27^, but since then only plastomes of *Fossombronia* *cristula*, *Makinoa* *crispata*, *Pallavicinia* *lyellii*^19^ and water form of *A.* *endiviifolia*^28^ have been sequenced. The mitogenomic resources of Pelliidae were limited to three species of Fossombroniales: *F.* *cristula*, *F.* *foveolata* and *M.* *crispata*^29,30^ and mitogenomes of orders Pelliales and Pallaviciniales remain unexplored.

Liverwort plastomes, as well as other plant plastomes, exhibit two copies of inverted repeats (IRa and IRb) separated by two single copy regions: the large single copy (LSC) and the small single copy (SSC). Unlike vascular plants, where there is a wide range of variation in plastome structure at the family or even genus level^31,32^, the gene content and gene order in the plastomes of liverworts are highly conserved across all major liverwort lineages, including complex thalloid, simple thalloid, and leafy species^19,33–35^. This consistency has been maintained despite millions of years of evolution separating these lineages^36^. The liverwort mitogenomes, unlike in tracheophytes, are characterised by stable structure and low number of repeats^37^ which play main role in mitogenomic rearrangements^38,39^.

In this study we sequenced, assembled and annotated 44 organellar genomes of Pelliidae, with special focus on Pelliales and their cryptic species complexes. Both organellar genomes were evaluated towards their super-barcoding potential.

## Material and methods

### Plant material

Plant material consisted of 22 fresh samples of *Pellia* s.l. collected from different geographical regions and different types of habitats and two herbarium specimens of *Fossombronia* *foveolata* and *Moerckia* *flotoviana*. The specimen and identification researchers details are given in Supplementary Table S1.

The cryptic species of the *Pellia* *epiphylla* complex were identified using peroxidase isozyme staining according to Zielinski methodology^22^. To confirm morphological identification of *P.* *borealis*, the number of chromosomes were counted for each specimen of *P.* *epiphylla* / *P.* *borealis* complex as described in earlier study^22^. Specimens with nine chromosomes were deliminated on either N or S based on presence different alleles in peroxidase locus. The samples with 18 chromosomes and peroxidase band pattern specific for *Pellia* *epiphylla* N were classified as *P.* *borealis*. Specimens with poor band staining or without confirmed chromosome numbers were excluded from analysis. For delimitation of *Apopellia* cryptic species intron–exon splice markers (ISJ) were used^24^ according to previously published protocol^40^. Experimental research and field studies on plants (either cultivated or wild), including the collection of plant material, complied with relevant institutional, national, and international guidelines and legislation.

### DNA extraction

DNA from the fresh samples was isolated by the modified CTAB procedure. The liquid nitrogen-ground thalii were thoroughly mixed with 3 ml preheated CTAB isolation buffer (2% CTAB, 100 mM Tris–HCl, pH 8.0, 20 mM EDTA, 1.4 M NaCl and 2% β-mercaptoethanol) and incubated at 55 °C for 1 h. After three chloroform extractions, the DNA was precipitated and dissolved in sterile, deionized H_2_O. Total genomic DNA from herbarium specimens was extracted using ZR Plant/Seed DNA MiniPrep™ kit (Zymo Research Corp., Irvine, CA, USA) according to the manufacturer’s protocol. The purity of DNA samples was assessed spectrophotometrically using Cary 60 spectrophotometer (Agilent). DNA quantity was estimated using the Qubit fluorometer and Qubit™ dsDNA BR Assay Kit (Invitrogen, Carsbad, NM, USA). DNA quality was checked by electrophoresis in 0.5% agarose gel stained with Euryx Simple Safe (Eurx, Gdańsk, Poland). The extracted DNA prior to long-read sequencing was carefully examined using Tapestation Genomic DNA kit (Agilent) and additionally cleaned using Genomic DNA Clean and Concentrator kit (Zymo, Irvine, USA).

### Library preparation, sequencing and assembly

The genomic libraries were constructed with DNA TrueSeq (Illumina) and were sequenced using HiSeqX (Illumina) to generate 150 bp paired-end reads at Macrogen Inc. (Seoul, Korea) with 350 bp insert in size between paired-ends. Afterwards, sequencing reads were cleaned by removing the adaptor sequences and low quality reads with Trimmomatic v0.36^41^. The long-read libraries were constructed using Ligation Sequencing Kit SQK-LSK109 (Oxford Nanopore Technologies) and NEBNext^®^ Companion Module for Oxford Nanopore Technologies^®^ Ligation Sequencing (New England Biolabs) according to manufacturer’s protocol and sequenced using MinION MK1B portable device (ONT) and R.9.4.1 Flow Cell (ONT). The Flow Cell was prepared for sequencing with Flow Cell Priming Kit EXP-FLP002 (ONT). Sequence reads were basecalled using high-accuracy (HAC) mode of Guppy 6.0.7 basecaller.

The previously sequenced Pellidae genomes, including plastome of *Apopellia* *endiviifolia* (NC_019628) and mitogenomes of *Fossombronia* *cristula* (MK645818) and *Makinoa* *crispata* (MK230958) couldn't be used as direct references due to low sequence similarity, especially considering *Pellia* sensu stricto genus. Therefore to assemble *Pellia* reference genomes we applied previously published approaches^42^. To assemble the *Fossombronia* *crispula* plastome SSR8246023 reads archive was used. The specimen details including GenBank accession numbers of newly assembled genomes are given in Supplementary Table S1.

The reference mitochondrial genomes for *Pellia* were assembled using Flye 2.8^43^ with default settings to overcome long regions of short tandem repeats. The remaining *Pellia* organellar genomes were assembled using NOVOplasty 4.3.1^44^. The GeSeq software was used for annotation of organellar genomes^45^ and genome maps were drawn using OGDRaw web server^46^. The junctions between chloroplast inverted repeats and single copy regions were visualised using IRscope software^47^.

Complete chloroplast and mitogenome genome sequences of 75 specimens, including 17 from Pellidae were used for phylogenetic analysis. Organellar genomes were initially aligned using MAFFT and ambiguously aligned regions were trimmed by Gblocks 0.91^48^. However, due to significant evolutionary divergence, reliable alignment wasn’t possible for most plastid intergenic and intronic regions, therefore for the main phylogenetic analyses only protein-coding genes (PCG) sequences were selected. The mitogenome dataset was also reduced to PCG, due to the loss of most intergenic spacers in genus *Apopellia* and short tandem repeat richness of *Pellia* intergenic spacers.

Phylogenetic analysis was carried out using the maximum likelihood (ML). The optimal model for the plastome dataset was identified as GTR + F + I + G4 by ModelFinder based on the Bayesian information criterion (BIC). ML analysis was performed using IQ-tree^49^. Mitochondrion and plastome trees were compared using cophylo function of phytools 1.0-3 R package^50^.

In order to explore plastome and mitogenome gene-tree conflict across liverworts phylogeny the single gene trees were calculated for each plastid and mitochondrial PCG.

The consistency among single genes was evaluated by the maximum-likelihood (ML) analysis for every locus that contained parsimony informative characters using the RAxML v7.2.3 plugin^51^ for Geneious Prime 2022 with the GTR + I + G model and bootstraps estimated using 1000 replicates. Potential conflicts among genes were visualised by constructing a supernetwork using SplitsTree4^52^ according to the approach given by Liu et al.^37^, except the number of replicates was increased to 1000 and the software used for creating consensus trees was Geneious Prime 2022. The supernetworks were created separately for chloroplast and mitochondrial datasets.

### Genetic variation, barcoding and molecular species delimitation

Comparative analysis of chloroplast genomes was carried out in the Spider 1.1-5 program^53^ based on inter- and intraspecific distances that were calculated using the Kimura 2-parameter model (K2P) of nucleotide substitution. Due to reasons mentioned above *Apopellia* and *Pellia* datasets were analysed separately. Barcoding analyses of entire *Pellia/Apopellia* organellar genomes and their 500 bp-long fragments generated by sliding window (step by 100 bp) were made in Spider. The discrete molecular diagnostic characters (MDCs) for each species were calculated according to the Jörger and Schrödl^54^ approach using FASTACHAR software^55^. Nucleotide diversity analyses were carried out using the PopGenome package^56^ with the window size and steps as in Spider analyses. Genetic similarity among *Apopellia* and *Pellia* species was calculated using simplot function of ggmsa package^57^, with sliding windows parameters as in previous analyses and accession numbers OL654073 (cpDNA), OQ236455 (mtDNA) and OQ280821 (cpDNA), OQ236469 (mtDNA) as references for *Apopellia* and *Pellia* respectively. Next to species delimitation analysis in Spider, ASAP: assemble species by automatic partitioning^58^ was applied. This method generates ten partitions, computing for each partition the asap-score, which is the average of two components: probability of panmixia (p-value) and barcoding gap width. The lower the score, the better the partition for the dataset. As input data alignments of plastomes and mitogenomes were used separately in each analysis.

### Codon usage analysis

The relative synonymous codon usage (RSCU) indicator was calculated based on the number of times a particular codon is observed, relative to the number of times that the codon would be observed assuming a uniform synonymous codon usage^59^. The RSCU values were calculated using the *seqinr* 4.2 R package^60^ and visualised using ggplot2^61^.

To calculate difference in codon usage bias between *Apopellia* and *Pellia* protein-coding genes we applied the Measure Independent of Length and Composition (MILC) with hard filtering option to remove sequences shorter than 80 AA, as implemented in coRdon 1.15.0 package^62,63^. Differences in codon usage (CU) between genera were visualised using intraBplot function of coRdon package^63^.

## Results

### Characteristics of Pelliidae chloroplast genomes

**Figure 1:**
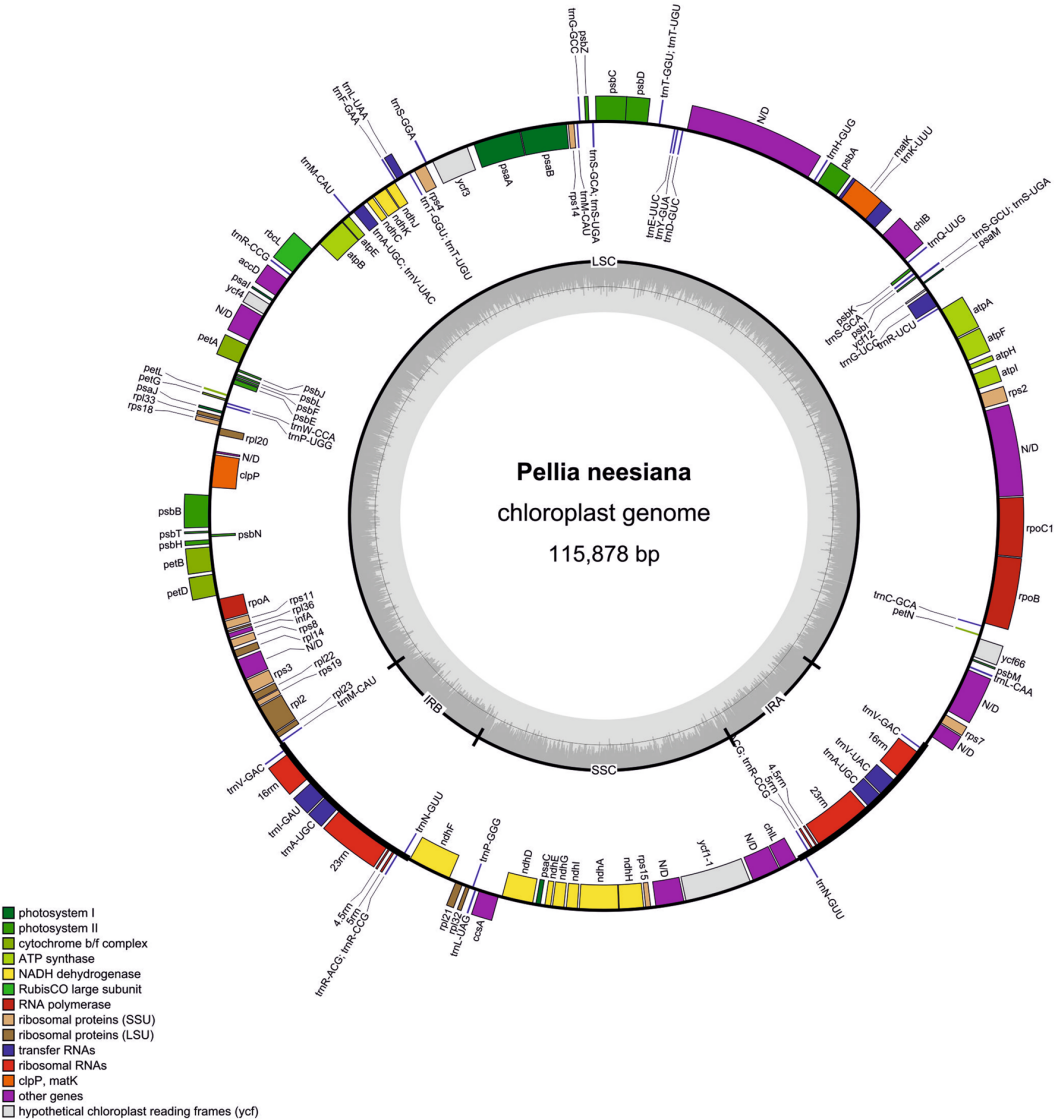
Gene map of the plastome of *Pellia* *neesiana*. Genes inside and outside the outer circle are transcribed in counterclockwise and clockwise direction, respectively. The genes are colour-coded based on their function. The inner circle visualises the GC content. * indicates genes containing introns.

Newly sequenced plastid genomes of Pelliidae are circular molecules consisting of regions typical for land plants. The total length of assembled plastomes ranged from 115,878 bp in *Pellia* *neesiana* (Fig. 1) to 124,598 bp in *Moerckia* *flotoviana*. The GC content ranged from 35.8% in water form of *A.* *endiviifolia* to 41.6% in *F.* *foveolata*.

One hundred twenty-two unique genes (taking into account only one copy of inverted repeat regions) were identified in the plastomes of *Apopellia* and *Moerckia*: 81 protein-coding genes, four ribosomal RNAs, 31 transfer RNAs and six *ycf* genes of an indeterminate function. The plastomes of *Fossombronia* and *Pellia* genera lack *cys*A and *cys*T genes, additionally in the *Fossombronia*, clusters of *trn*S(GCU)-*psb*I-*trn*S(GCA) genes are missing. The gene order seems to be conserved in Pelliidae, with exception of translocation of *rps*12-*rps*7-*ndh*B to the minus strand of LSC/IR boundary.

### Plastid genome variation

**Figure 2:**
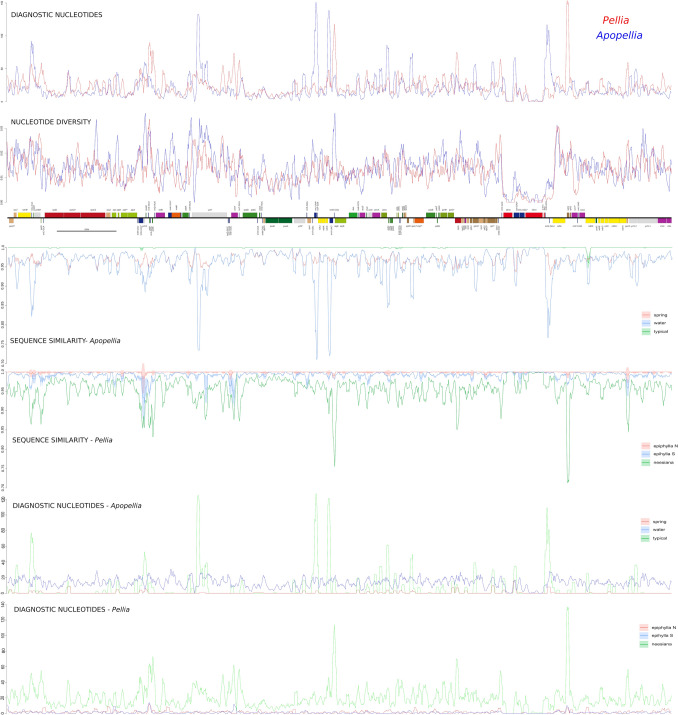
Comparison of diagnostic nucleotides, sequences similarity and nucleotide diversity between plastome of *Apopellia* and *Pellia* genera together with intrageneric specificity of *Pellia* (spring, water and typical) and *Apopeliia* (N, S, and nessiana) specimen.

The nucleotide diversity (Pi) values of 500 bp long frames across plastomes varies from 0 in rRNA genes of IR regions to 0.034 in *ycf*12-*trn*Q and *trn*V-*atp*E intergenic spacers (igs) (Fig. 2).

The values within IR regions were almost five times lower than within single copy LSC and SSC parts of the plastome. In the both genera the single copy regions near LSC-IRb and IRb-SSC regions were also highly variable, especially in the case of SSC, where *ndh*F gene is located. The second peak of nucleotide diversity within the SSC region was within the *ycf*1 gene. However, the gene near the SSC-IRa border, *chl*L, was among less variable regions of *Pellia* and *Apopellia* plastomes.

The patterns of nucleotide diversity within *Apopellia* and *Pellia* genera usually overlap with few additional hotspots of the former found in the *rpo*C2 gene and igs between *atp*I-*atp*H, *psb*M-*trn*S, *psb*A-*trn*H and *trn*V-*atp*E (Fig. 2). Lower variation in *Apopellia* than *Pellia* was revealed at 5’ end of *ycf*2, across whole *acc*D, *rpl*22 and *chI*L genes.

The analysis of sequence similarity within the same 500 bp window revealed some regions of intraspecific variation, which was generally higher in *Pellia* than *Apopellia* (Fig. 2). In the case of the latter slight variation at cryptic species level was found within the *ndh*F gene and the *trn*G(UCC)-*ycf*12 spacer of the typical form. The mentioned spacer was also variable within *P.* *epiphylla* N and S. These two species shared infraspecific variation within few other plastome regions including *ndh*B-*ycf*66, *atp*H-*atp*F, *ycf*2-*cys*A, *ndh*C-*trn*V and *ndh*H-*ycf*1b. Additionally, plastomes of *P.* *epiphylla* S revealed variation in the *ycf*2 gene, *ycf*3-*rps*4 region and between *rrn*12 and *rrn*23 genes of IR.

### Characteristics of Pelliidae mitochondrial genome

**Figure 3:**
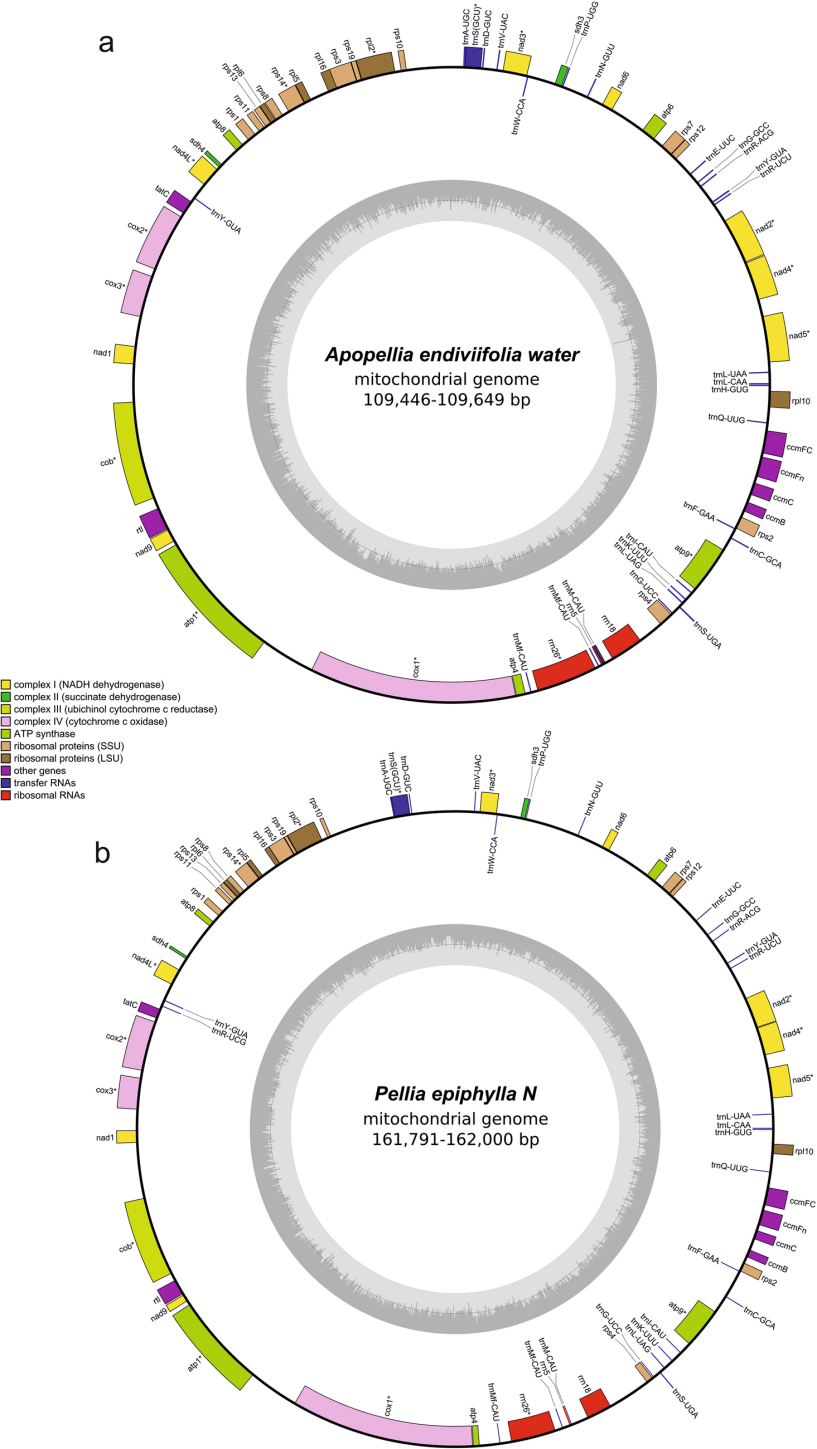
Mitochondrial genomes of (**a**) *Apopellia* *endiviifolia* water form and (**b**) *Pellia* *epiphylla* N. Genes inside and outside the outer circle are transcribed in counterclockwise and clockwise direction, respectively. The genes are colour-coded based on their function. The inner circle visualises the GC content. * indicates genes containing introns.

The newly assembled mitochondrial genomes have almost identical gene content and order, as previously published (MK230958, MK749462 and MK230936) with exception of the *trn*R-UCG loss in the *Apopellia* genus (Fig. 3a). The mitogenomes of Pellidae ranged from 108,928 bp in typical form of *Apopellia* *endiviifolia* to 179,862 bp in *Moerckia* *flotoviana* (Table S1), but the length of the molecule doesn’t correlate with intron content (Fig. 3b).

**Figure 4:**
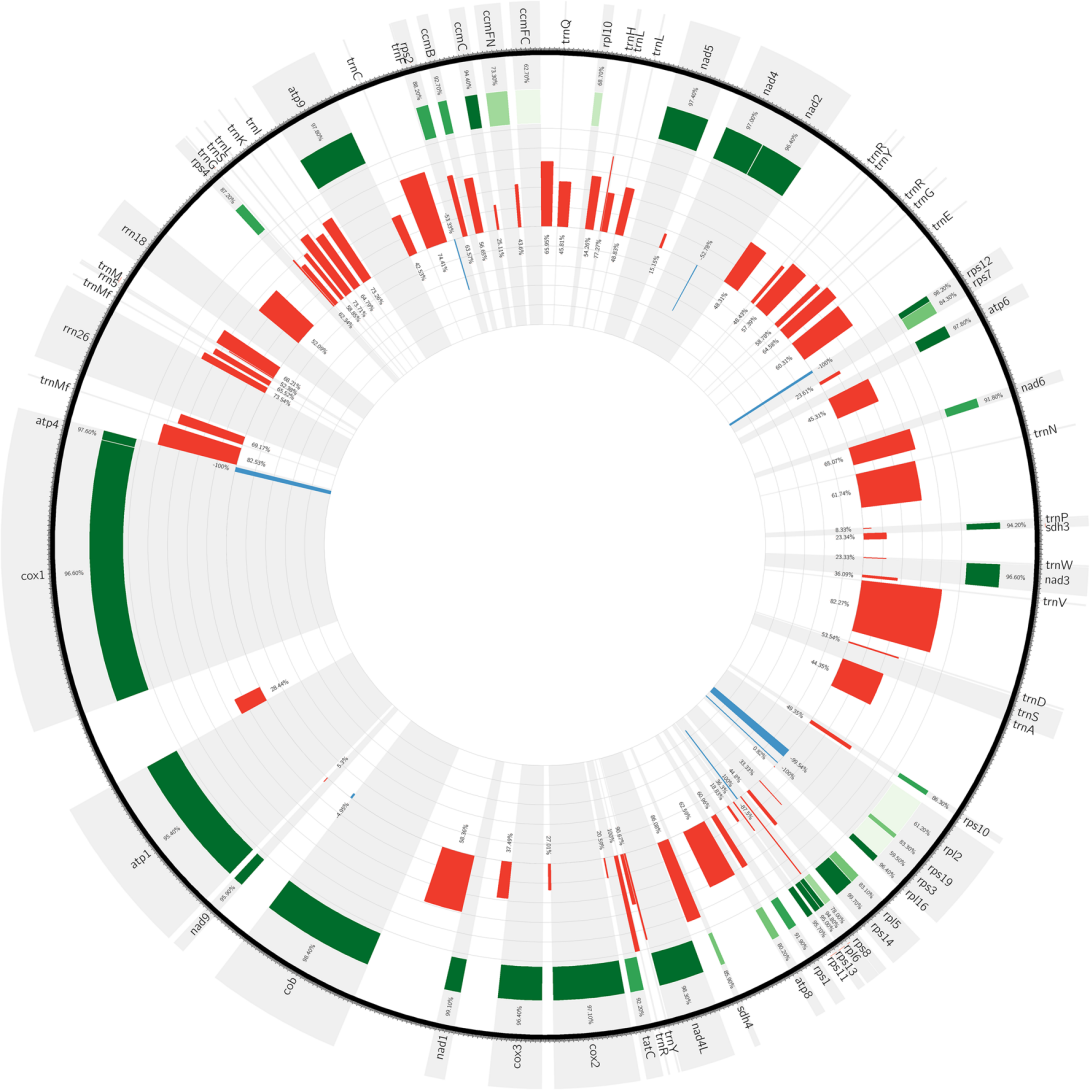
Circular visualisation of difference in spacers length between *Pellia* and *Apopellia*. Red and blue histograms represent percentage differences in intergenic spacers sequences. Height of red bars show increasing ratio length for *Pellia* versus *Apopellia* and width indicate direct dissimilarity between sequences length of both species. The blue bars show *Apopellia* advantage in length. The green heatmap depicts pairwise alignment of each coding region between both species.

The species of Fossombroniales order lack both introns of the *atp*1 gene, and in the genus *Fossombronia* lost two introns of *cox*1, being the second and third largest mitogenomes of Pellidae (179,676 bp and 175,222 bp respectively). In contrast, over 70 kbp smaller mitogenomes of *Apopellia* contain complete introns set. The reduction of *Apopellia* mitogenome in comparison to the closest related *Pellia* is due to the loss of nucleotides in intergenic regions (Fig. 4). The intergenic spacers like *atp*4-*trn*Mf, *trn*V*-trn*D, *sdh*4-*nad*4L, *nad*4L-*tat*C were over 80% shorter, while in the most of igs reduction fall within 40–60% range. Among the larger igs the smallest reduction, by 28.4% were in the *atp*1-*cox*1. Seven very short intergenic spacers were longer in *Apopellia* than in *Pellia* and in the one longer between *cob*-*nad*9 genes with the difference lower than 5%. Despite extraordinary reduction of igs, the identity between genes remains high, mostly above 90% (Fig. 4).

### Mitochondrial genome variation

**Figure 5:**
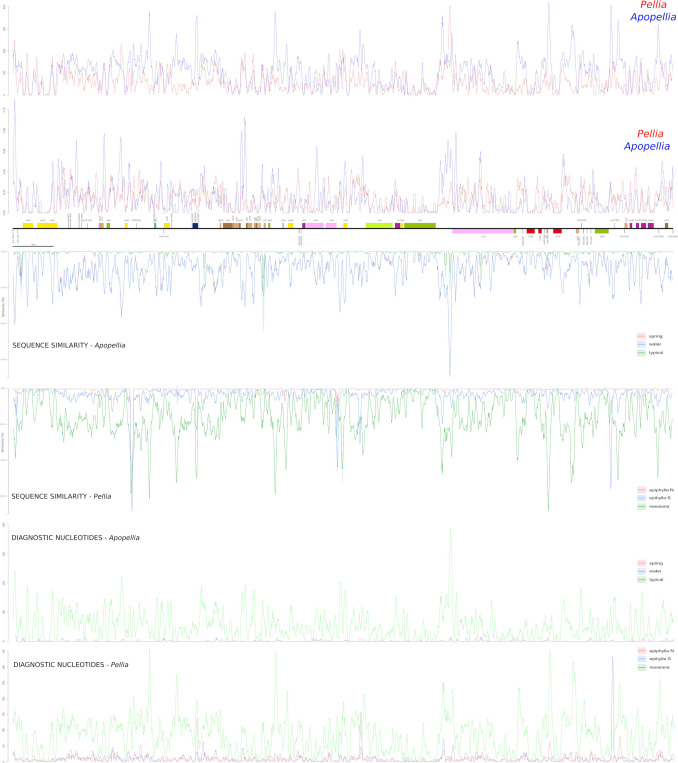
Comparison of diagnostic nucleotides, sequences similarity and nucleotide diversity between mitogenome of *Apopellia* and *Pellia* genera together with intrageneric specificity of *Pellia* (spring, water and typical) and *Apopeliia* (N, S, and nessiana) specimen. Sliding window analyses of *Apopellia* and *Pellia* mitogenomes is equal to 500 bp.

The nucleotide diversity (Pi) values of 500 bp-long frames across mitogenomes ranged from 0 in frames located along protein-coding genes (like *nad*4 and *nad*5) to 0.11 in *Apopellia* *trn*L(CAA)-*trn*L(UAA) igs (Fig. 5). The Pi values were generally lower in *Pellia*, with the highest peak (0.048) revealed within *ccm*B-*ccm*C igs. Beside this spacer, only five regions of *Pellia* mitogenome had values exceeding 0.04 including the following igs: *nad*2-*trn*R(UCU), *trn*Y(GUA)-*trn*R(ACG), *trn*N(GUU)-*trn*P(UGG), *nad*4L-*trn*Y(GUA) and *nad*1-*cob*. In *Apopellia* dataset, 22 regions had Pi values higher than 0.04, and six of them exceeded 0.06: *trn*Y(GUA)-*trn*R(ACG), *rps*7-*atp*6, *atp*6-*nad*6, *rpl*16-*rpl*5, intron of *cox*2 and *atp*1-*cox*1. The intraspecific variation of mitochondrial genomes of both genera was detected only within typical form of *A.* *endiviifolia* and *P.* *epiphylla* S. In the latter only two variable regions were revealed: *trn*L(CAA)-*trn*L(UAA) and *trn*V(UAC)-*trn*D(GUC). The variation among individuals of typical *A.* *endiviifolia* was identified in 22 regions, but the top five most variable regions (with pairwise similarity below 0.8) were *trn*L(UAA)-*nad*5, *trn*A(UGC)-*rps*10, *rps*11-*rps*1, *atp*1-*cox*1 and middle introns of *cox*1.

### Organellar phylogenomics of liverworts with special focus on subclass Pellidae

**Figure 6:**
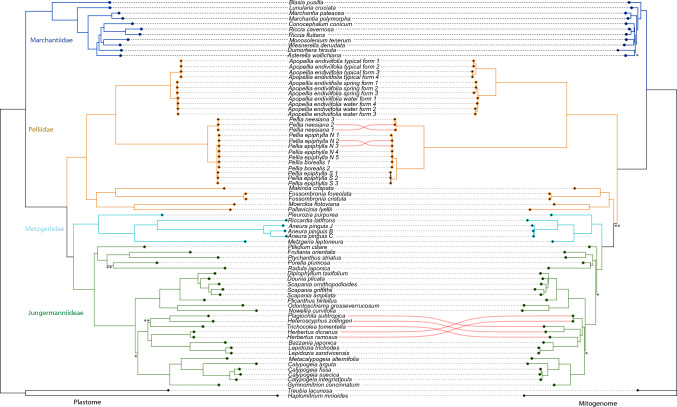
Phylogenetic topology inferred from concentrated plastid (left) and mitochondrial dataset (right) using ML method. All clades are maximally supported unless indicated otherwise with double asterix (for BS values within 85–99 range) or single asterix (for BS values below 85). Clade positions were optimised using cophylo function in the phylotools R package.

The concentrated chloroplast gene dataset consists of 64,602 nucleotides including 39,885 variable and 34,191 parsimony informative genes. The plastid phylogeny recovered three major liverwort lineages: Haplomitriopsida (outgroup), Marchantiopsida and Jungermanniopsida (Fig. 6). Within the Jungermanniopsida all three subclasses: Pellidae, Metzgeriidae and Jungermanniidae were resolved as monophyletic groups with maximal bootstrap support. The species of subclass Pellidae formed two main clades (with maximum BS support): one grouping specimens of the order Pelliales and the second formed by species belonging to Fossombroniales and Pallaviciniales. The latter was splitted into subclades congruently with order affiliation: *Makinoa* and *Fossombronia* formed a common clade as members of Fossombroniales, while *Moerckia* and *Pallavinicia* were grouped together as genera of Pallaviciniales. The order Pelliales further splits into two clades corresponding to genera *Apopellia* and *Pellia*. Within the former, all cryptic lineages including typical, water and spring forms were resolved as monophyletic clades with maximum bootstrap support. The water habitat lineages (water and spring) form a common clade sister to the typical form. The second clade of Pelliales were formed by species of the genus *Pellia*, revealing *P.* *neesiana* as early divergent within this group. Cryptic species of *P.* *epiphylla* formed monophyletic, maximally supported groups with a common clade. Allopolyploid species *P.* *borealis* was included in *P.* *epiphylla* N clade.

The concentrated mitochondrial gene dataset consists of 45,852 nucleotides including 18,257 variable and 13,051 parsimony informative. The mitochondrial dataset generated a tree with similar, but not identical topology as the plastid dataset. Analysis of mitochondrial genes didn’t support monophyly of Pelliidae and resolved Pelliales as earliest divergent among Jungermanniopsida, however support for common Fossombronialles/Pallaviciniales—Jungermanniideae clade wasn’t maximal (BS 91%), while the plastid dataset supported monophyly of Pellidae with maximum value. Another significant difference was revealed within suborder Lophocoleineae where species of *Herbertus* were placed as the earliest divergent. (Fig. 5). Minor differences comprise relationships within complex thalloid liverworts: (1) the mitochondrial dataset placed *Asterella* and *Dumortiera* within common clade (but poorly supported—BS 69%); (2) relationships at intraspecific level within *Pellia* *neesiana* and *P.* *epiphylla* N.

The supernetworks (Supplementary Fig. 1) based on single-gene trees of the mitochondrial and plastid dataset didn’t reveal significant phylogenetic conflicts among genes, especially during diversification of the major liverwort lineages (Fig. 6). The competing splits within Pelliidae appeared only in the *Pellia* clade, where some minor incongruences in the mitochondrial dataset were detected among *Pellia* *epiphylla* N and *P.* *neesiana* lineages.

### Codon usage differentiation of *Apopellia* and *Pellia*

**Figure 7:**
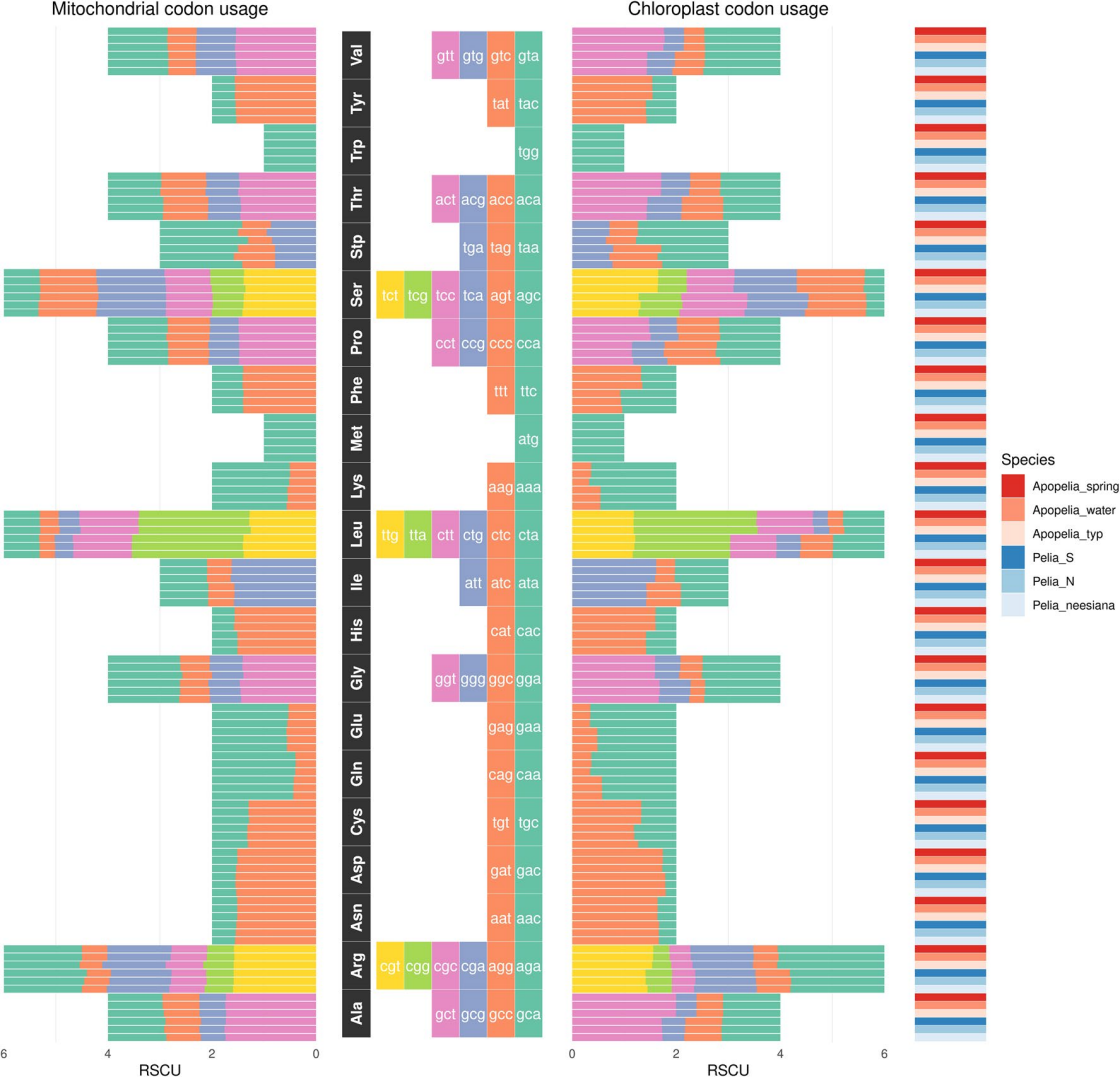
Codon usage of mitochondrial and chloroplast genomes for six selected species of *Pelliidae*. The main histograms (left—mitochondrial, right—chloroplast) depict RSCU values for each amino-acid`s coding triplets based on MILC method.

Comparative codon usage analysis revealed a difference between *Apopellia* and *Pellia* plastome genes, despite sharing by these genera identical sets of plastid *trn*S (Fig. 7). However, in the case of mitochondrial protein-coding genes, the CU patterns of both genera were similar and the loss of one *trn*R(ugc) gene by *Apopellia* mitogenomes didn’t impact CU. The differences in codon usage among *Apopellia* and *Pellia* aren’t equally distributed among amino acids (Supplementary Fig. 2, Fig. 7). In the *Apopellia* plastomes most common stop codon ‘taa’ is preferred over ‘tag’, while in *Pellia* all stop codons have similar frequency. Patterns of CU for amino acids like tyrosine, glutamine, glutamate, asparagine, aspartate and cysteine were similar in both genera. Where amino acids were coded by more than two codons, the differences in CU usually comprised two of them, like in the case of alanine, where ‘gct’ codon is more frequent than ‘gcc’ in *Apopellia*, while the opposite situation was revealed in *Pellia.*

The general pattern of CU in the mitogenome and plastome was similar (Fig. 7) and as mentioned above, without clear differences between studied genera. Within *Apopellia* differences were found in ‘water’ form, in which serotonine ‘tcg’ and ‘tct’ codon frequencies are different than in remaining species. This kind of variation was also found in CU of leucine and arginine amino acids, where ‘water’ form of *A.* *endiviifolia* revealed different CU than *Pellia* and *A.* *endiviifolia* typical and spring forms.

### Barcoding and MDCs

The number of detected plastid MDCs in *Apopellia* and *Pellia* genera varied from 0 to 4717 (Supplementary Table 2). No MDCs were identified for allopolyploid *Pellia* *borealis*, as two analysed specimens fell within variation of *Pellia* *epiphylla* N. Therefore plastomes of *P.* *borealis* were excluded from barcoding and molecular delimitation analyses. Among other species, the lowest number of MDCs (136) was detected for the spring form of *A.* *endiviifolia* followed by *P.* *epiphylla* S with and *P.* *epiphylla* N with 235 and 393 MDCs respectively. The highest numbers of MDCs were revealed for *P.* *neesiana* (4717) and for typical form of *A.* *endiviifolia* (3161). The impact of multiple specimen sampling on the number of detected MDCs, with the exception of the *P.* *epiphylla* complex, was minimal, below 1% decrease in MDCs. In the case of *P.* *epiphylla* S the number of MDCs decreased by 31% (mean for individuals 342 vs 235 at species level) and 15% for *P.* *epiphylla* N (mean for individuals 463 vs 393 at species level).

The number of detected mitochondrial MDCs in *Apopellia* and *Pellia* genera varied from four to 30,538 (Supplementary Table 2). The lowest number of MDCs was revealed for *P.* *borealis*, while the highest for *P.* *neesiana*. The cryptic species of the *P.* *epiphylla* complex have 2979 and 3307 MDCs for N and S respectively. Among *Apopellia* *endiviifolia* cryptic species the biggest number of MDCs was identified for the typical form (20,260), while the water and spring form revealed 309 and 264 MDCs respectively.

Comparison of infra- and interspecific distances based on the plastome confirmed the presence of a significant barcoding gap among all species, including cryptic ones (Supplementary Fig. 3). In the case of mitochondrial genomes, the barcoding gaps are smaller but still significant. The share of nucleotide diagnostic characters is not equally distributed among analysed species (Fig. 2).

**Figure 8:**
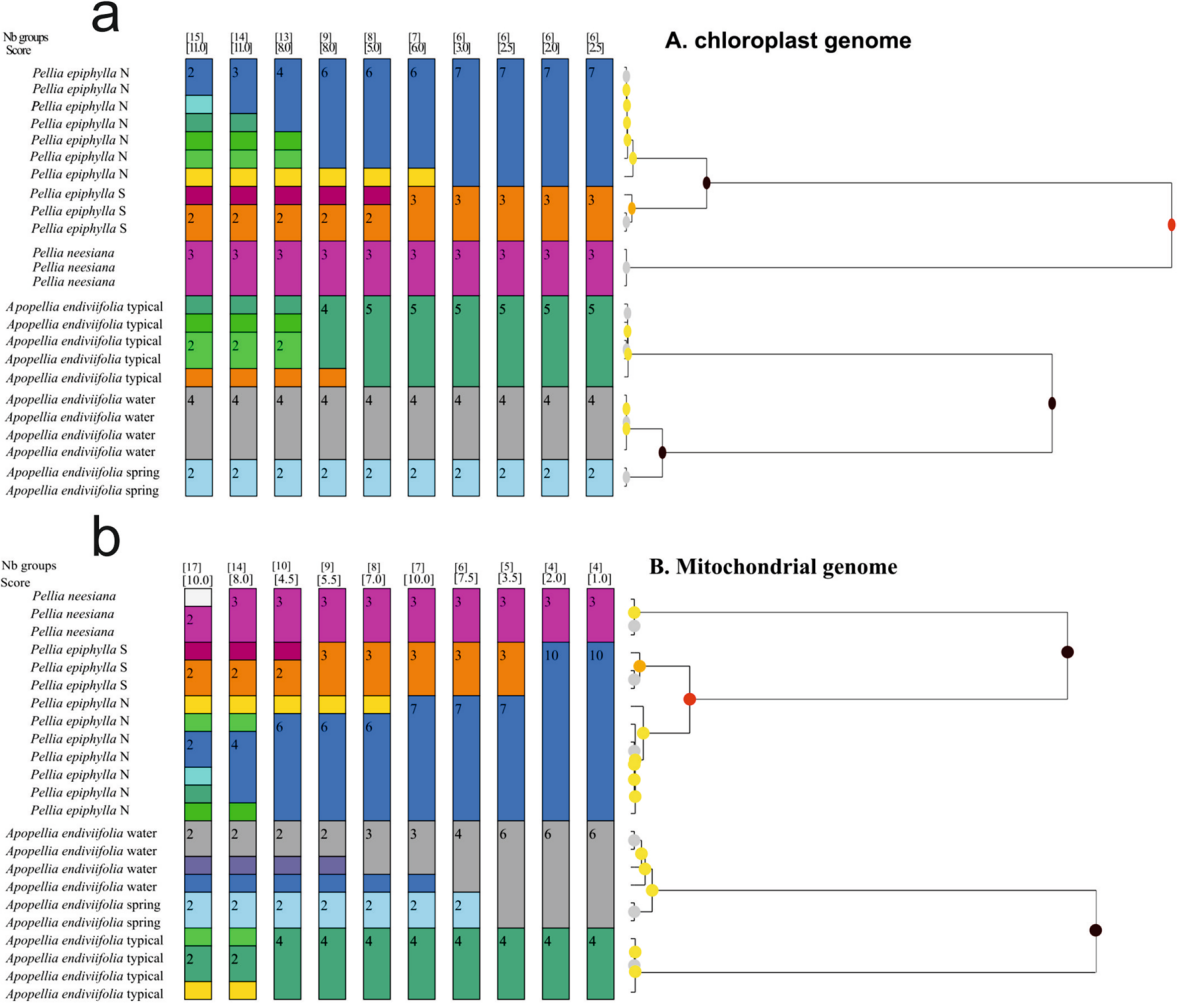
Species delimitation by ASAP analysis for chloroplast (**a**) and mitochondrial (**b**) genomes. The colorful fields indicate the groups of species. Every field contains the number of individuals. Above the coroful bars the coefficient asap-score (the lower value) and number of species (the upper value) recognized for the whole dataset are presented. The cladogram supports the species delimitation presented on the bar charts.

ASAP analysis performed for data based on the chloroplast genome generated four identical partitions with low values of asap-score ranging from two to three (Fig. 8a). These four proposed divisions indicated six species among testing liverwort groups. These results fully reflect the species split among the cryptic complexes in the genera *Pellia* and *Apopellia*. Within the *Pellia* genus, cladogram indicated that *P.* *epiphylla* N and *P.* *epiphylla* S are definitely separated species (black circle, Fig. 8a). As different species, cladogram also clearly showed two forms of *Apopellia* *endiviifolia*: water and spring (black circle, Fig. 8a).

The mitogenome-based dataset gave slightly weaker results. Only one partition (asap-score = 7.5) proposed by ASAP was congruent with real division of *Pellia* and *Apopellia*, indicating a total of six species within these genera (Fig. 8b). On the other hand, one partition with quite low asap-score with a value of 3.5 grouped together only water and spring form of *Apopellia*. Although two ASAP-partitions put forms of *Pellia* *epiphylla* together, the cladogram next to the bar chart suggests that *Pellia* *epiphylla* is not a homogeneous group and the division into N and S types is justified (red circle, Fig. 8b).

## Discussion

### Organellar genomes of Pellidae

The liverwort organellar genomes are known from its conserved structure and gene content and results of Pelliidae analysis confirm this observation. In contrast to the chloroplast genomes of angiosperms, the structural heteroplasmy connected with SSC subunit orientation wasn’t detected in Pellidae, as well as in previous studies on leafy liverwort *Scapania* *undulata*^64^. Long read sequencing identified only one plastome haplotype also in gymnosperms and pteridophytes, suggesting that alternative haplotype is specific for angiosperms^65^. The gene order of Pelliales and Fossombroniales is typical for most of liverworts, but plastome structure of *Moerckia* confirms that the translocation of *rps*12-*rps*7-*ndh*B cluster seems to be characteristic for Pallaviciniales^19^.

Plastomes of the *Pellia* genus lost two genes (*cys*A and *cys*T), but gene order remains stable. These genes are present in the sister genera *Apopellia,* *Makinoa* *and* *Moerckia*^19,28^, but in the remaining genera of Pelliidae (*Fossombronia*, *Pallavicinia,* *Pellia*) are pseudogenised, as well as in many other leafy liverworts^19^. Further gene losses include *trn*S(GCU)-*psb*I-*trn*S(GCA) in *Makinoa* and *Fossombronia*. However, plastome length in Pellidae doesn’t correlate with gene losses, while the second largest plastome of *Pallavicinia* (124,103 bp) and the smallest of *Pellia* *neesiana* (115,875 bp) have identical gene content, whereas plastomes of *Apopellia* with complete gene sets (120,537–120,947 bp), are smaller than *Pallavicinia* without *cys*A and *cys*T genes. Independently of gene loss, plastome reduction was also found in leafy Cephaloziineae, which plastomes ranged between 118,571 and 114,423 bp, despite identical gene sets^28,64^. Plastid gene losses are scattered along different evolutionary lineages including loss of *cys*A and *cys*T genes in *Ptilidium* *pulcherrimum* and *Cheilolejeunea* *xanthocarpa*^33,35^ and additional four genes (*ndh*F, *rpl*21, *rps*32 and *ccs*A) in *Cololejeunea* *lanciloba*^66^. Single genes are also missing in *Aneura* *pinguis* (*ycf*66), *Haplomitrium* *blumei* (*psa*M) and *Schistochila* *macrodonta* (*ndh*F)^66,67^. Heterotrophic *Aneura* *mirabilis* has a reduced plastome due to loss of six genes (*ndh*A-E-G-K-I and *ycf*66) or pseudogenization (19 genes) of genes involved in the process of photosynthesis. Two liverwort species increase the number of plastid genes by transferring them to IRs: *Haplomitrium* *blumei* transferred *ndh*F gene from SSC^66^, while *Conocephalum* *salebrosum* transferred *rps*7 and *rps*12 from LSC^68^. Minor changes in genomes are related to loss of introns: complex thalloid liverworts, with the exception of Blasiales, lost introns of *ycf*3 and *rps*12 genes, which phenomenon was also reported in species from distantly related genera like *Herbertus*, *Ptychantus*, *Radula* and *Metzgeria*^19,35,68^. Single loss of introns of *ndh*A and *ndh*B genes were found in *Radula* *japonica* and *Treubia* *lacunosa*^19^.

In contrast to plastomes, evolutionary losses of mitochondrial introns seem to be common in liverworts, but mainly concern leafy liverwort lineages, while the intron content of thallus liverworts is stable^29,30,69^. Out of ten intron losses only the loss of *trn*L could happen once and remaining losses were independent across different evolutionary lineages of leafy liverworts^70^. Unlike previously sequenced genomes of Pelliidae (*Fossombronia* *cristula* and *Makinoa* *crispata*), the mitogenomes of *Pellia* and *Apopellia* have introns in the *atp*1 gene and in contrast to *Fossombronia*, keep complete *cox*1 intron set^30,70^. The gene content of liverwort mitogenomes is more stable than that of the plastomes, beside the *nad*7 loss, which is present only in early divergent lineages of Haplomitriales and Treubiales and the loss of *ccm*C and *ccm*Fn in *Treubia* *lacunosa*^71^. The variation of tRNA genes among liverworts comprises the loss of trnT-GGU after divergence Marchantiopsida and Jungermanniopsida and *trn*R-UGC after divergence of Pellidae. The *trn*R-UGC seems to be independently lost in *Apopellia*, even considering incongruent results on monophyly or paraphyly of Pelliae^14,66,70,72^, which doesn't question monophyly of *Apopellia*/*Pellia* clade^14,26^. The loss of the *trn*R-UGC gene didn’t impact codon usage pattern of mitochondrial protein coding genes—both genera revealed similar CU pattern (Figs. 7, 8). On the other hand, *Apopellia* and *Pellia* differ in CU by plastome CDS, despite identical *tRNA* gene content. The lack of correlation between codon usage and the number of tRNA was previously reported in bacterial genomes^73,74^. Procaryotes seem to respond to gains or losses of tRNA genes by modulating the concentration of tRNAs rather than modifying its frequency on codon usage^73^.

Despite the presence of some repeats, which are considered as a main factor of mitochondrial structure dynamics in vascular plants^75,76^, the structure of liverwort mitogenomes is stable^37^. The exceptions were found for the mitogenome of *Gymnomitrion* *concinnatum* belonging to Jungermanniales, where two recombination events were detected^34^ and in *Dumortiera* *hirsuta* (Marchantiales) where one 96 kb inversion was found^77^. The possibility of recombination was also suggested for 12 other liverworts, however most frequent variants support typical mitogenome structure^29^.

Despite the nearly identical gene content (loss of *trn*R-UCG in *Apopellia*) and order the mitogenomes of *Pellia* and *Apopellia* differ in size. The newly sequenced *Apopellia* mitogenomes were smallest among known liverworts, over 49 kbp smaller than those found in *Pellia* and falling within 109,022–109,828 bp in range. The size of *Apopellia* mitogenomes is closer to the values found in mosses^37,78,79^, than to those known in liverwort mitogenomes ranging from 142,510 bp in *Tritomaria* *quinquedentata* to 187,628 bp in *Monosolenium* *tenerum*^29,69^. The size reduction of *Apopellia* mitogenome is not caused by gene or intron losses, but by the reduction of intergenic spacers, which affects all regions with exception of only few spacers like *cob*-*nad*9, *cox*3-*nad*9 and usually long *atp*1-*cox*1, which was reduced only by 31%. The reduction or increase of mitogenome length in leafy liverworts is often caused by indels between genes *nad*3 and *rpl*10. This region contains the pseudogenized (with exception of *Haplomitrium* and *Treubia* where *nad*7 is functional) *nad*7 gene and three tRNAs: *trn*V-UAC, *trn*D-GUC, *trn*S-GCU and is a main source of mitogenome size variation among *Nowellia* and *Scapania* species^28^. Comparative analyses of Pelliidae mitogenomes also confirmed this observation revealing over 11 kbp difference in *nad*3-*rpl*10 region between *Makinoa* and *Fossombronia* species, with differ by 8 kbp in total mitogenome length (*Makinoa* contains two more *cox*1 introns). This region is also twofold smaller in *Apopellia* (5.3 kbp) than in *Pellia* (11.4 kbp), but it comprises only 11% of total mitogenome reduction in the former.

Phylogenetic placement of Pelliidae based on plastid sequence is mostly congruent with previous studies^28,35,70^. One minor difference is the position of *Conocephalum* *conicum*, which in the work of Dong et al. was resolved as an unusual clade, probably due to plastome misassembly (GB accession number MK645816). The latest study on complex thalloid phylogeny confirms the position of *Conocephalum* as sister to *Riccia*^80^. Chloroplast dataset supports monophyly of Pelliidae, however the mitogenome dataset resolved Fossombroniales and Pallaviciniales in common clade with remaining Jungermanniopsida, while Pelliales was resolved as the earliest divergent from that class. Cyto-nuclear incongruences are common across phylogeny of vascular plants^81^ and were also confirmed within Pellidae, where the application of transcriptomic data revealed that mito-phylogenetic results support nuclear gene topology^19^. Incongruences among nuclear, mitochondrial and plastid dataset are often explained by incomplete lineage sorting^82^, horizontal gene transfers^83^ and hybridization events^84^, however in the case of Pellidae aforementioned mechanisms are unlikely. The organellar genomes of Pellidae seem to evolve much faster than any other subclass of liverworts.

The topology of the obtained trees is congruent with previous studies on Pelliales and fully supports splitting of *Pellia* sensu lato by placing *Pellia* *endiviifolia* in the separate genus *Apopellia*^26^. The splitting of the *Pellia* genus is not only supported by typical sequence phylogenetic divergence but also by differences in the structures and genome gene content as discussed above. Moreover, the mitochondrial dataset revealed a paraphyly of Pelliaceae and resolved *Apopellia* as a sister group to Fossombroniaceae.

### Super-barcoding and molecular diagnostic characters

Application of organellar genomes as super-barcodes enabled identification of all analysed evolutionary lineages of *Pellia* s.l. with the exception of allopolyploid *P.* *borealis* which inherited organellar genomes from *P.* *epiphylla* *N*^85^.

The number of plastid MDCs characterising *Apopellia* species decreased in comparison to previous study^28^, due to expanding the dataset by a spring form, which reveals some intermediate characters between water and terrestrial forms, but it still, like *P.* *epiphylla* S and N, falls within the range known from *Calypogeia*—a taxonomically good liverwort species^86,87^.

Based on our data it is hard to estimate the effect of multiple specimen sampling on presence of diagnostic nucleotides or MDCs. In four out of six species (including cryptic) the impact of sample number within 2–6 range on plastid MDCs was below 1% (species of *Apopellia* and *P.* *neesiana*). The decrease in plastid MDCs by 31% was detected in *P.* *epiphylla* S (N = 3) and by 15% in *P.* *epiphylla* N (N = 6), but it was not correlated with sample size. The mitogenomes reveal contrasting patterns, but again, not correlated with sample size. The decrease in MDCs in the case of *P.* *epiphylla* cryptic species was 7% and 9% for S and N respectively, but in the case of *A.* *endiviifolia* water form decrease in MDCs was 35%.

The mitogenomes deliver more MDCs than the plastome, however two factors should be discussed. The number of detected MDCs depends on alignment size, which in the case of mitogenomes was as much as 71% longer, however it doesn’t explain the over 5.6-fold higher number of MDCs. In this particular case, the main reason for the outnumber of MDCs in mitogenome is a bigger number of large indels in mitochondrial dataset (1385 indels with mean length of 146.2 bp) in comparison to plastid dataset (1629 indels of 9.1 bp mean length). Thus the mitogenomes were more efficient in delimitation of cryptic species within the *P.* *epiphylla* complex, while plastomes performed better in the case of *A.* *endiviifolia* complex. These results can be explained by differences in genome sizes, since the mitogenome of *Apopellia* is smaller than plastome, which is quite unusual in plants, with exception of mosses^37,78,88^.

Comparative organellar genomics support previous hypotheses about allopolyploid origin of *Pellia* *borealis*, which pointed out *Pellia* *epiphylla* N as a maternal species^85^. Both organellar genomes found in *P.* *borealis* were almost identical to those found in *P.* *epiphylla* N. The lack of plastome MDCs and only four MDCs in mitogenomes suggest the late Pleistocene origin of *P.* *borealis*, when ranges of the N and S lineages could form a natural hybridization zone. The allopolyploid *Calypogeia* *muelleriana*, which carries the maternally inherited plastome of *C.* *integristupula*, revealed specific SNPs enabling molecular delimitation of this species based on the plastome and mitochondrial datasets^86,87^.

The distribution of *Pellia* *epiphylla* cryptic species can’t be described as northern (N) and southern (S) lineages and their ranges should be revisited. Previous studies revealed geographical vicariance despite similar ecological requirements^22,89^, but nowadays we can’t confirm this pattern, since N lineage was also found in Carpathians. Coexistence of cryptic species lineages is common in other known complexes as *Aneura* *pinguis*^3^ or *Apopellia* *endiviifolia*^22^.

Our results confirm the presence of the third cryptic lineage named “C” within *Apopellia* *endiviifolia*, described previously by Polok et al.^24^ as “well-headed” form. Phylogenetic analyses resolved species C as a sister to A which corresponds to similar ecological preferences of these forms. However, based on current knowledge on distribution of species C, its habitat preferences are much narrower than in the case of species A. All known specimens of species C occurred directly in the springs, they cover the bottoms of forming watercourses. The earlier studies suggested that species A is known mainly from the mountains in the direct neighbourhood of running water, with very restricted occurrence in the lowlands, which corresponds to preferred microhabitat distribution^90^. The sites with clear and fast running shallow water, which this lineage depends on, are more common in the mountains. The geographic distribution of species B is concentrated in lowlands with rare occurrence in the mountains and the species prefers denuded soil and rock detritus^90^. The species C is currently known from five sites, including three lowland and two mountain locations^24^ and its scattered distribution can be explained by presence of spring areas. Morphological studies on *A.* *endiviifolia* revealed significant differences between cryptic species in quantitative traits like size of basal and marginal cells, even after six months of greenhouse culture^24^. The species C had the smallest basal and marginal cells within *A.* *endiviifolia* complex. However, further ecological and morphological studies are needed to better characterise cryptic species within *A.* *endiviifolia* which could lead to formal taxonomic recognition. A species called *Apopellia* *megaspora* was identified in the early 1980s as one of the cryptic North American species of *A.* *endiviifolia*^91^. In addition to differences in spore diameters, it is distinguished from other *Apopellia* species by the absence of thallus proliferation in autumn. Another cryptic species of simple thalloid liverwort, *Aneura* *pinguis*, has been more thoroughly studied, but despite the presence of various lineage-specific DNA markers^3,67,92^ and many biochemical compounds^93^, it lacks significant morpho-anatomical differentiation. Furthermore, the cryptic lineages of *A.* *endiviifolia* and some of *A.* *pinguis* differ in their preferred microhabitats, which can be characterised not only by general ecological descriptions, but also by specific parameters such as pH and concentrations of calcium, magnesium, potassium, and sodium^94^.

Results presented in this study provide novel insights on evolution of liverwort organellar genomes, revealing high differentiation of mitogenomes among closely related taxa. Both plastid and mitochondrial genomes proved their usefulness in molecular delimitation of cryptic species of *Apopellia* and *Pellia*. Surprisingly, effectiveness of mitogenomes in species discrimination within Pellidae was on a similar level as plastome.

## Supplementary Information

Supplementary Information.

## Data Availability

The datasets used and/or analysed during the current study available from the corresponding author on reasonable request.
