# Supplementary figures and images for: The organellar genomes of Pellidae (Marchantiophyta): the evidence of cryptic speciation, conflicting phylogenies and extraordinary reduction of mitogenomes in simple thalloid liverwort lineage
Source: Sci Rep. 2023 May 23;13:8303. doi: 10.1038/s41598-023-35269-3 (PMC10205812; doi:10.1038/s41598-023-35269-3)

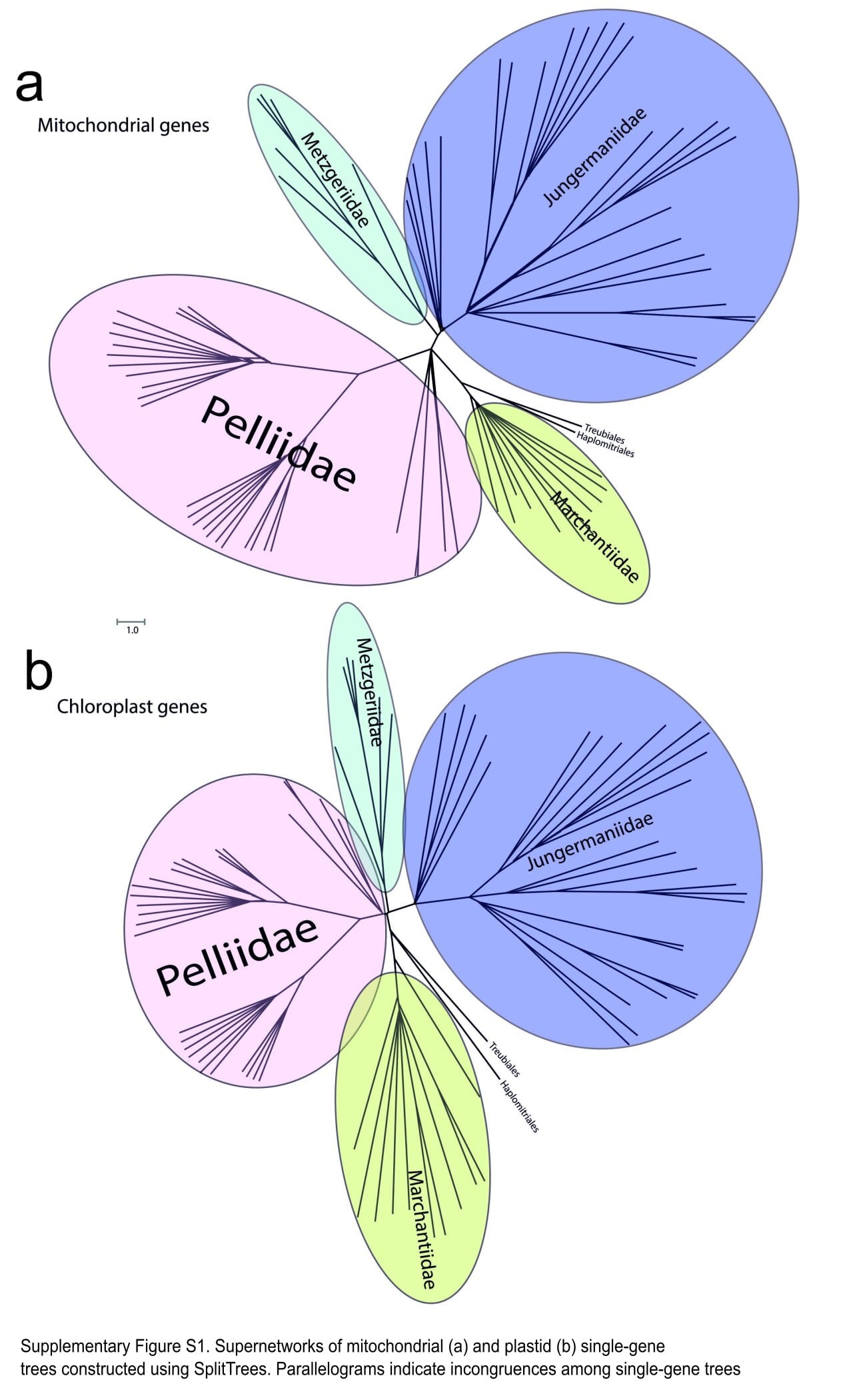

Supplement: Supplementary file 1 — Supplementary Information. [file 41598_2023_35269_MOESM1_ESM.zip › Supplementary Figure 1.jpg]

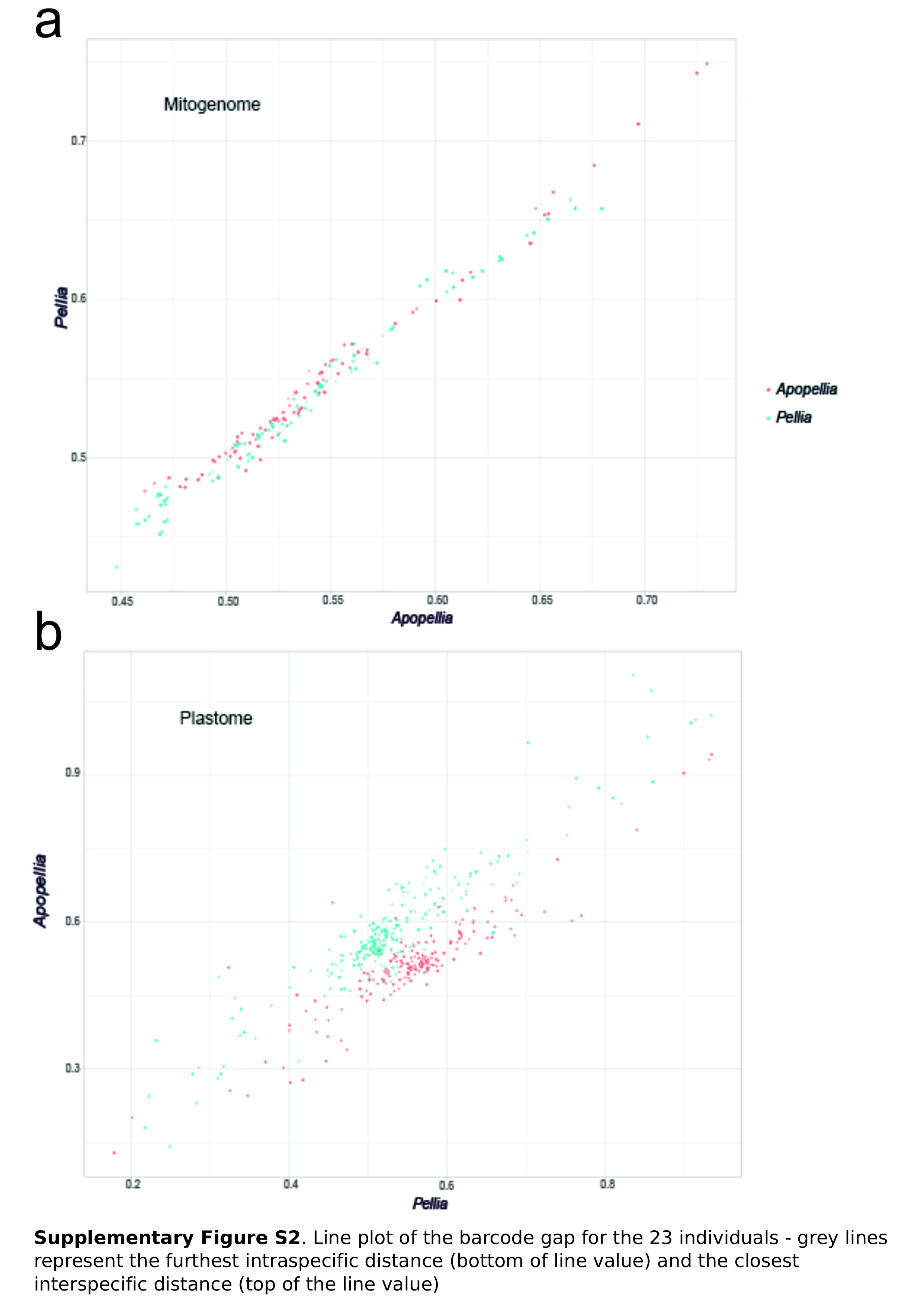

Supplement: Supplementary file 1 — Supplementary Information. [file 41598_2023_35269_MOESM1_ESM.zip › Supplementary Figure 2.jpg]

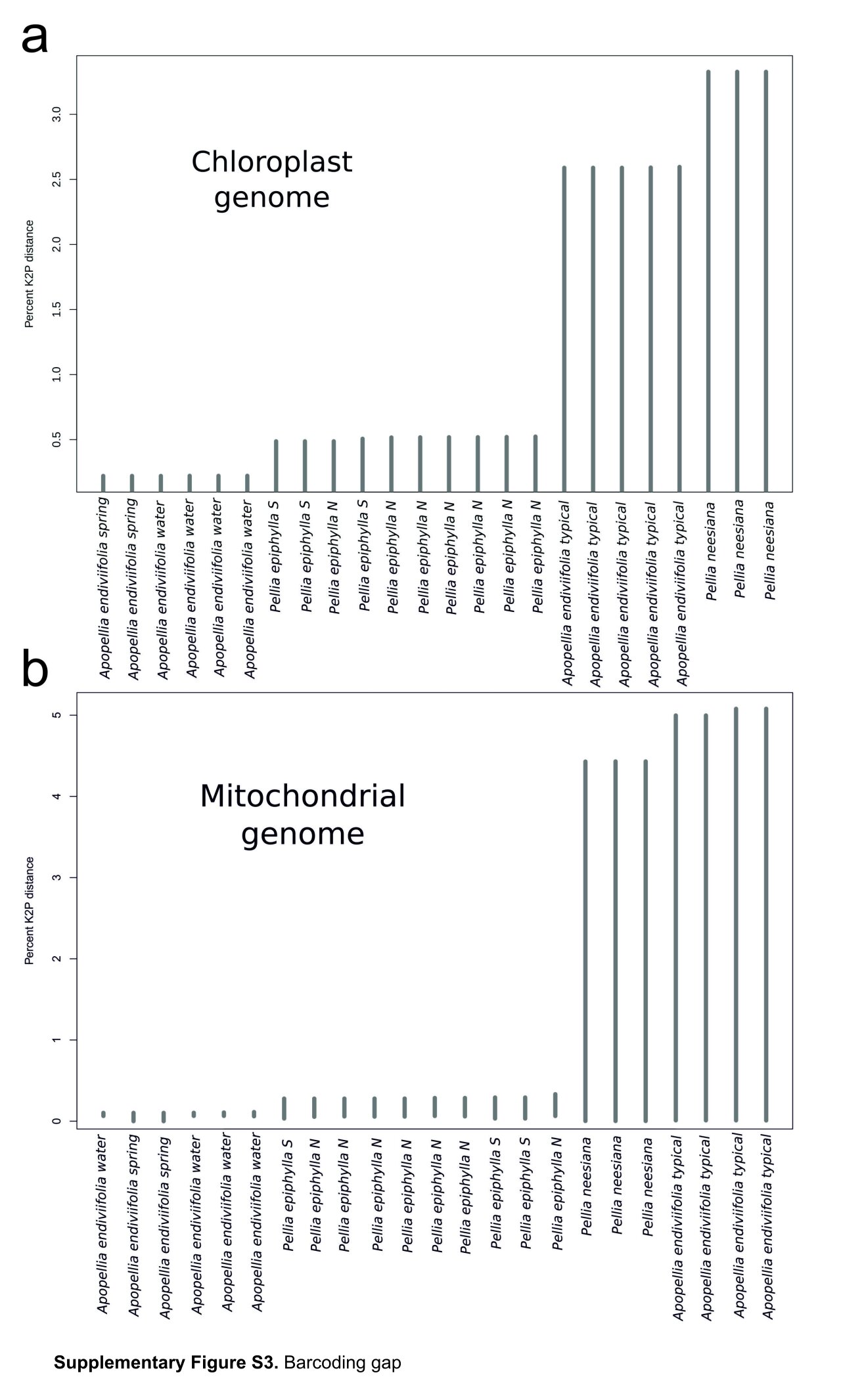

Supplement: Supplementary file 1 — Supplementary Information. [file 41598_2023_35269_MOESM1_ESM.zip › Supplementary Figure 3.jpg]
